# Proton-conducting polymer electrolyte membranes based on sulfonated PEEK and blends of protic ionic liquids with different acidities

**DOI:** 10.1038/s41598-025-22147-3

**Published:** 2025-10-09

**Authors:** V. Theußl, X. Cui, H. Hou, A. Gueguen, C. Rodenbücher, C. Korte

**Affiliations:** 1 Institute of Energy Technologies – Electrochemical Process Engineering (IET-4), Forschungszentrum Jülich GmbH, 52425 Jülich, Germany; 2 Institute of Physical Chemistry, RWTH Aachen University, Landoltweg 2, 52074 Aachen, Germany; 3 Toyota Motor Europe BV Technical Centre, Hoge Wei 33, 1930 Zaventem, Belgium

**Keywords:** Electrocatalysis, Fuel cells

## Abstract

Protic ionic liquids (PILs) immobilised in an ionogene host polymer represent an intriguing alternative to the state-of-the-art commercial fluoropolymer-based membranes as proton conducting electrolytes for future intermediate-temperature fuel cells (T > 100 °C, IT-PEMFCs). In this study, two PILs, *N*, *N*-diethyl-3-sulfopropane-1-ammonium trifluoromethanesulfonate [DESPA][TfO] and diethyl-methylammonium trifluoromethanesulfonate [DEMA][TfO], were investigated for their suitability as proton conducting species in a sulfonated poly(ether-ether-ketone) membrane (sPEEK). Both PIL membrane casts exhibit good thermal stability until 200 °C and a high ionic conductivity of up to 5.28 mS.cm^−1^(120 °C, 40%RH). The polarisation curves showed a maximum power density of 3.5 mW.cm^−2^ for [DESPA][TfO] membrane cast and 6.5 mW.cm^−2^ for [DEMA][TfO] membrane cast, proving the suitability of the PIL membrane cast as a polymer electrolyte membrane (PEM) for the IT-PEMFCs.

**Supplementary Information:**

The online version contains supplementary material available at 10.1038/s41598-025-22147-3.

## Introduction

Polymer electrolyte fuel cells (PEMFCs) are an intriguing technology for clean power production in place of fossil fuel-based generation, as their utilisation can be employed in almost every application where local electricity generation is needed^1^. Especially in the transportation sector, PEMFCs constitute a viable alternative to internal combustion engines, capable of powering zero-emission vehicles operated with sustainable fuels such as hydrogen, produced by utilising renewable energies^2,3^. In the long run, vehicles based on fuel cells are even more promising than rechargeable lithium batteries, especially for heavy-duty transport, due to their much lower weight and less required space. Short recharging/refilling times within several minutes and a much higher operational range will allow for greater autonomy, in contrast to battery-driven vehicles^4–6^.

State-of-the-art PEMFCs that utilise perfluorosulfonic acid-based membranes, *e.g.*, Nafion^®^ or Aquivion^®^, however, rely on certain restrictions. Perfluorosulfonic acid-based membranes require sufficient hydration for good proton conductivity. This restricts the operating temperature to < 80 °C. With higher temperatures (> 100 °C), simplified water management due to reduced dependency on membrane hydration and the potential for waste heat recovery would be enabled in addition to a decreased sensitivity of the Pt-catalyst to impurities in the feed gas^7–11^. High-temperature PEMFCs, with an operating temperature of 160–180 °C, however, exhibit a low power density, which can be attributed to the sluggish ORR kinetics resulting from catalyst poisoning by phosphate ions^12^. With the intermediate temperature PEMFC (> 100 °C), a possible compromise solution is obtained as the intermediate temperature combines the advantages of both systems without the need for phosphoric acid as a conducting electrolyte. However, developing suitable membranes for intermediate operating temperatures remains a key challenge. Due to a lack of suitable materials, it is still necessary to proceed with the search for alternative materials capable of maintaining high proton conductivity and chemical stability to close the “conductivity gap” between the commercially available PEMs for implementing the intermediate temperature PEM fuel cells^13^.

Ionic liquids have attracted attention in recent years as promising candidates for use as conductive electrolytes in PEMs for future IT-PEMFCs. Compared to their commercially available counteract, perfluorosulfonic acid (PFSA) membranes, membrane casts doped with ionic liquids have the significant advantage of good proton conduction even in anhydrous or low-humidity environments. Furthermore, ionic liquids’ low volatility and high thermal stability enable the maintenance of structural integrity and performance even under high-temperature conditions^14–17^. Protic ionic liquids (PIL) are especially interesting in this respect, as they possess a high proton conductivity and offer a wide electrochemical window^18,19^. Implementing these novel PILs in an IT-PEMFC requires immobilising the ionic liquids in a mechanically stable membrane to sustain stable conductivity and mechanical stability. Different preparation methods are possible, from a diffusional uptake of the PIL by the polymer (swelling process)^20,21^to solution casting methods^22,23^, to direct polymerisation of PIL monomers^24^. The solution casting method seems to be the most effective as it allows uniform distribution of the PIL and an easy adjustment of its concentration^25^. Concerning the polymer matrices used, polybenzimidazole (PBI) is the most studied polymer matrix for immobilization due to its application in HT-PEMFCs with phosphoric acid^26–29^. Apart from PBI, other polymer matrices, such as sPEEK, can also be implemented. sPEEK has the significant advantage of being a low-cost polymer material. Next to its thermal stability and mechanical properties, it additionally holds intrinsic proton donor moieties through the sulfonation process and enhanced hydrophilicity^30^.

Based on the results of preceding studies on the electrode kinetics of PILs, we have chosen two ILs with different proton donor abilities, *i.e.* acidity, as a higher proton donor ability results in more negative ORR onset potentials^31^. The chosen ILs were diethyl-methylammonium trifluoromethanesulfonate [DEMA][TfO], as a low acidity PIL, and N, N-diethyl-3-sulfopropyl-1-ammonium trifluoromethanesulfonate [DESPA][TfO], a high acidity PIL. The higher the acidity and the higher the proton donor ability, the more negative ORR onset potentials should be obtained^31^. [DEMA][TfO], as low acidity PIL, generally shows higher conductivity, better O_2_ solubility and -diffusivity, leading to higher diffusion limiting currents. Furthermore, it shows a higher hydrophobicity and a higher overvoltage, represented by a more negative onset potential. On the other hand, [DESPA][TfO], the PIL with the higher acidity, shows a lower conductivity in the anhydrous state, but as soon as water is present, higher conductivities than [DEMA][TfO] are obtained. The O_2_ solubility and -diffusivity, however, are lower than in the case of [DEMA][TfO], leading to lower diffusion limiting currents^31,32^. Moreover, [DESPA][TfO] have lower overvoltages, representing a more positive onset potential. Discussing the further components of the composite membrane, sPEEK was selected due to its hydrophilic properties and its intrinsic good proton conductivity. PBI is less hydrophilic, and its intrinsic conductivity in the case of water swelling is very low. In addition, the proton conduction of sPEEK is similar to that of Nafion^®^. Apart from this, sPEEK as a fluorine-free polymer also provides high thermal-, mechanical- and chemical stability, as well as has a good film-forming ability^33,34^. To circumvent possible membrane degradation due to excessive swelling at high humidification and in the presence of a liquid water phase, the sPEEK membrane was also crosslinked via PEG. The crosslinking was achieved via intra/inter-chain ester-condensation of the reactive sulfonic acid functions, initiated by a thermal treatment^35,36^. Regarding the amount of PEG, in previous tests, we found that the amount of PEG had an impact on the ionic conductivity up to a certain weight%. Figure S1 shows the obtained results of this pre-study in dependence on the relative humidity, which showed an increase in conductivity up to a mass percentage of 3 wt% PEG.

This study investigated novel PIL membrane casts doped with ionic liquids as conductive electrolytes to evaluate their suitability for future IT-PEMFCs. The investigated ionic liquids, [DESPA][TfO] and [DEMA][TfO], are immobilised in a sulfonated polyether ether ketone (sPEEK) membrane matrix. For the determination of their suitability, full cell test measurements are performed. Additionally, thermal stability is evaluated using thermal gravimetric analysis (TGA) and ionic conductivity by electrochemical impedance spectroscopy (EIS) measurements. Scanning electron microscopy (SEM) was carried out to investigate the morphology of the membranes.

## Experimental section

### Fabrication of PIL membrane casts

**Fig. 1:**
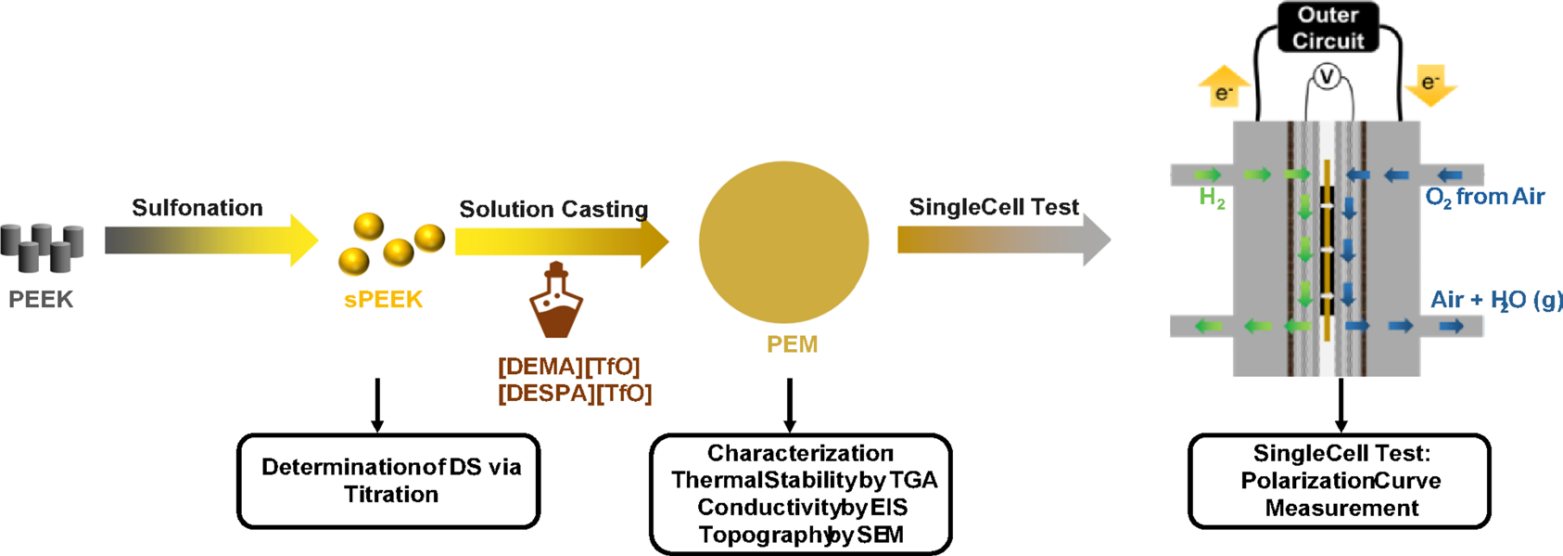
Experimental flowchart of the preparation and characterisation steps of the prepared membranes.

Certain steps are involved in fabricating PIL/sPEEK composite membranes, including sulfonation of the host polymer polyether ether ketone (PEEK) and incorporation of the PIL into the host polymer *via* solution casting. The host polymer, sulfonated polyether ether ketone (sPEEK), was prepared through the post-sulfonation of dry PEEK pellets (15 g, PEEK 450G, Victrex, UK) using concentrated sulfuric acid (300 ml, 98%, Merck KGaA, Germany). For the sulfonation process, the mixture (PEEK and H_2_SO_4_) was heated to 45 °C and stirred for 6 h. The sulfonation process was terminated by cooling the solution in an ice bath (T < 10 °C), followed by pouring the solution into cooled deionised (DI) water. This results in a precipitation of the desired product, *i.e.* sPEEK. The obtained polymer particles were washed with DI water until a neutral pH was achieved and dried in an oven using a defined temperature program: 40 °C to 80 °C with a heating rate of 10 °C/24 hours and an isothermal step at 80 °C until a constant weight was obtained. The drying process was accompanied by a colour change of the polymer particles from white to yellow (as depicted in Fig. S1). An overview of the entire preparation and characterisation method is illustrated in an experimental flowchart shown in Fig. 1.

The success of the sulfonation process was confirmed by checking the obtained degree of sulfonation (DS), *i.e.* the percentage of PEEK repeating units with an SO_3_H moiety, see Eq. (1). Thus, a pH-titration was implemented to assess the ion exchange capacity (IEC) of the polymer that depends on the DS, see Eq. (2) and consisted of 4 steps: acid treatment, washing, ion exchange and titration. For the acid treatment, 0.2–0.3 g of dried sPEEK polymer was immersed in 1 M HCl for 24 h to fully protonate the sulfonic acid groups of the polymer. At the beginning of the acid treatment, the suspension was shaken in an orbital shaker (Vortex, IKA Werke, Germany) at 1000 rpm for 10 min. Next, the polymer was washed with DI water to remove excess acid and again dried at 80 °C under a vacuum until a constant weight ($\:{m}_\mathrm{sPEEK}$) was reached. For the ion exchange process, to replace the protons with Na^+^ ions, the dried sPEEK polymer was immersed for 24 h in 2 M NaCl and shaken in an orbital shaker (Vortex, IKA Werke, Germany) at 1000 rpm for 10 min initially. For the titration, the solution containing the released protons from the ion exchange process was separated from the polymer particles *via* filtration (200 ml). The number of released protons $\:{n}_{{\text{H}}^{+}}$ from the sPEEK were determined by titration with NaOH, which gave a measure of the total number of sulfonic acid groups present in the polymer. The titration (Titrando 888 with Stirrer 801, iUnitrode with Pt1000 from Metrohm) was repeated three times. The DS can be determined by the following Eq. (1):

1 $$\:\begin{array}{c}DS=\frac{IEC\cdot\:{M}_{\text{P}\text{E}\text{E}\text{K}}}{1-IEC\cdot\:{M}_{\text{S}{\text{O}}_{3}}}\end{array}$$

Thereby, $\:{M}_{\text{P}\text{E}\text{E}\text{K}}$ (g.mol^−1^) denotes the molar mass of the repeating unit of PEEK, $\:{M}_{\text{S}{\text{O}}_{3}}$ is the molar mass of SO_3_ (g mol^−1^), IEC is the determined ion exchange capacity from Eq. (2) (mol g^−1^). The IEC was determined using the following Eq. (2):

2 $$\:\begin{array}{c}IEC=\frac{{V}_{\text{N}\text{a}\text{O}\text{H}}\bullet\:{c}_{\text{N}\text{a}\text{O}\text{H}}\bullet\:{t}_{\text{N}\text{a}\text{O}\text{H}}}{\frac{50\:\text{m}\text{L}}{200\:\text{m}\text{L}}\bullet\:{m}_{\text{s}\text{P}\text{E}\text{E}\text{K}}}=\frac{{V}_{\text{N}\text{a}\text{O}\text{H}}\bullet\:{c}_{\text{N}\text{a}\text{O}\text{H}}\bullet\:{t}_{\text{N}\text{a}\text{O}\text{H}}}{\frac{1}{4}\bullet\:{m}_{\text{s}\text{P}\text{E}\text{E}\text{K}}}\end{array}$$

The concentration of NaOH used for titration is denoted by $\:\:{c}_{\text{N}\text{a}\text{O}\text{H}}$(0.05 M), the calibration coefficient by $\:{t}_{\text{N}\text{a}\text{O}\text{H}}$ (0.9682) and the volume of NaOH needed for titration by $\:{V}_{\text{N}\text{a}\text{O}\text{H}}$.

**Fig. 2:**
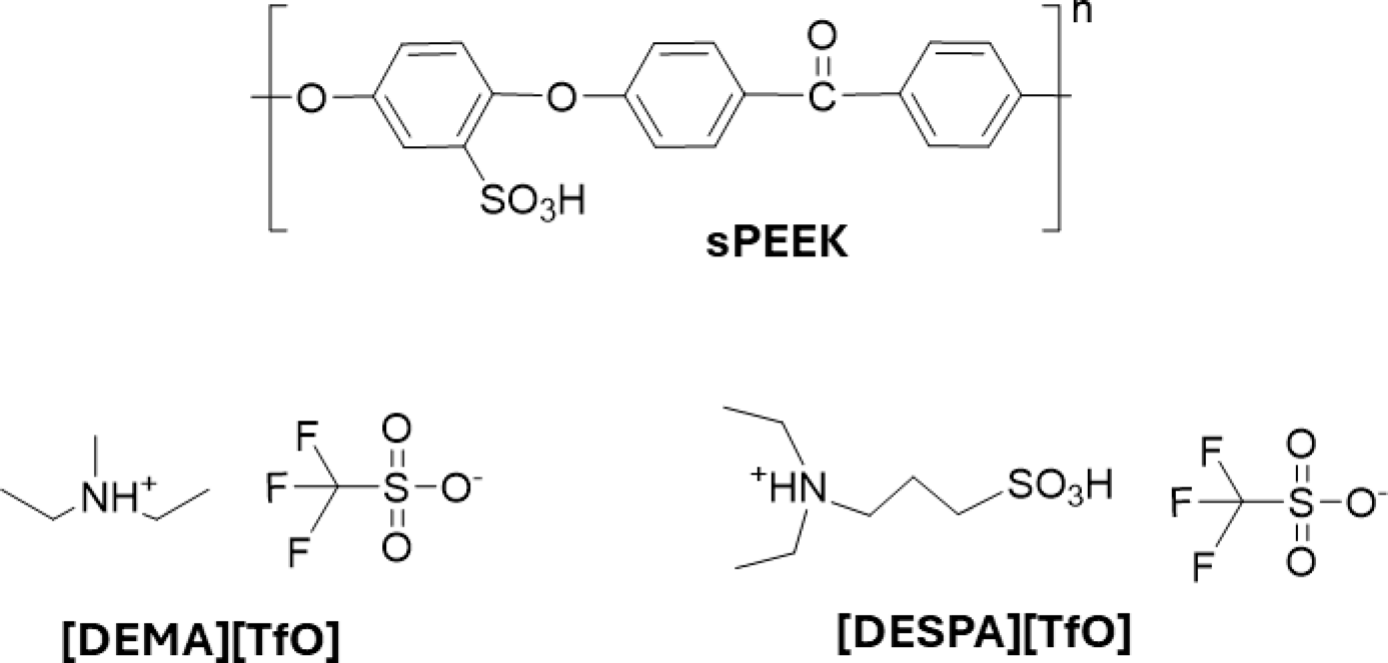
Structural formula of sPEEK and the PILs [DEMA][TfO] and [DESPA][TfO] utilised for the membrane preparation.

For the preparation of PIL/sPEEK membranes, a solution casting method was used. For this, the sPEEK polymer was dissolved in dimethyl sulfoxide (DMSO, for synthesis, Merck KGaA, Germany) at a 1:50 ratio. For the crosslinking process, 0.03 g polyethylene glycol (PEG 200, for synthesis, Merck KGaA, Germany) and 2 g of PIL were dissolved in 5 ml DMSO, either [DESPA][TfO] (> 98%, IoLiTec GmbH, Germany) or [DEMA][TfO] (> 98%, IoLiTec GmbH, Germany), were added to the sPEEK solution and stirred overnight. For illustration, the structural formulas of sPEEK and the PILs is shown in Fig. 2. To ensure thorough mixing, the solution was sonicated in an ultrasonic bath for 4 h and then cast on a Petri dish. To evaporate the solvent, the Petri dish was placed in an oven for 5 days (at 50 °C for 3 days and at 80 °C for 2 days). For the crosslinking process, the obtained membrane was dried again in a vacuum oven at a pressure below 10 mbar for 20 h at rising temperatures (at 80 °C for 2 h, at 100 °C for 2 h and at 140 °C for 16 h). The obtained PIL/sPEEK membranes had a 66 wt% loading of PIL, a degree of sulfonation of 50 ± 4% and a thickness of 116 ± 7 μm for [DESPA][TfO] and 168 ± 27 μm for [DEMA][TfO].

### Preparation of membrane electrode assemblies

For the electrochemical investigation of the PIL/sPEEK membranes, membrane electrode assemblies (MEA) were assembled. The MEAs consisted of two commercially available gas diffusion electrodes (GDE, Dapozol^®^ 300 Electrode, Danish Power Systems) with an active area of 17.64 cm^2^ and a Pt loading of 0.9 mg.cm^−2^. The PIL/sPEEK membranes with an area of 30.25 cm^2^ were sandwiched in between. The fusion of the MEA assembly was obtained by the generated pressure during the cell assembly (torque wrench: 2 N m, 4 N m, and 6 N m). The whole measurement setup is shown in Fig. S5.

### Characterisation of the PIL/sPEEK membranes

The thermal stability of the prepared PIL/sPEEK membranes ([DESPA][TfO] and [DEMA][TfO]) and its constituents (PEEK, sPEEK, PIL) was determined by thermal gravimetric analysis (TGA, STA 6000, Perkin Elmer Inc., USA) under a N_2_ atmosphere. The sample weight was 5–10 mg, and the temperature program is shown in Fig. S2. To counteract the effect of the temperature program on the sample pan, a background measurement with an empty sample pan at the same temperature program was conducted and subtracted from the sample results.

The (total) ionic conductivity of the PIL/sPEEK membranes was determined using an impedance analyser (1260, Solartron Analytical, UK) inside a climate chamber (WK3600/40, Weiss Technik GmbH, Germany). The operating conditions were *T* = 80–120 °C and a relative humidity of (%RH) = 10–40%. Membrane strips (10 mm × 40 mm) were placed on two parallel platinum wires, as depicted in Fig. S3, with a separation of 10 mm. The EIS measurements were performed in a frequency range of 1–10^7^ Hz and an excitation voltage amplitude of 50 mV. The conductivity of the membrane $\:{\sigma\:}_{\text{P}\text{E}\text{M}}\:$was calculated with the following Eq. (3):

3 $$\:\begin{array}{c}{\sigma\:}_{\text{P}\text{E}\text{M}}=\frac{l}{{R}_{\text{P}\text{E}\text{M}}\times\:w\times\:d}\:\end{array}$$

Thereby, $\:l$ denotes the length (distance between the two electrodes), $\:w$ the width and $\:d$ the thickness of the investigated PIL membrane casts. $\:{R}_{\text{P}\text{E}\text{M}}\:$denotes the resistance of the PIL/sPEEK membranes obtained during the EIS measurement.

For the determination of the temperature dependence of the ionic conductivity and to determine the activation energy $\:{E}_{\text{a}}$of the ionic conductivity, the Arrhenius equation is applied (4)^37^:

4 $$\:\begin{array}{c}\sigma\:=\frac{{\sigma}_{0}}{T}\mathrm{exp}\left(-\frac{{E}_{\text{a}}}{kT}\right)\end{array}$$

Thereby, $\:\sigma\:$ denotes the ionic conductivity, $\:{\sigma}_{0}$ the pre-exponential factor, $\:T$ the (absolute) temperature and $\:k$ the Boltzmann constant.

The activation energy $\:{E}_{a}$ can be obtained from the temperature dependent conductivity data by plotting $\:\text{ln}\left(\sigma\:T\right)$ vs. $\:\frac{1}{T}$ *via* the slope $\:-\frac{{E}_{a}}{k}$, see Eq. (5):

5 $$\:\begin{array}{c}\text{ln}\left(\sigma\:T\right)=\text{ln}{\sigma}_{0}-\frac{{E}_{a}}{k}\cdot\:\frac{1}{T}\end{array}$$

For the study of the PEM morphology, cross-sections of the cut PEMs were embedded in resin and investigated with a scanning electron microscope (SEM, Zeiss Ultra Plus FEG, Carl Zeiss AG, Germany). The SEM was operated at 5 kV, and the images were recorded at 2500x magnification.

The electrochemical characterisation was conducted in a fuel cell test rig (Greenlight Innovation, Canada) with a custom-made fuel cell. The fuel cell components (end plates, flow fields, MEA, and gaskets) were assembled with eight bolts by a torque wrench (2 N m, 4 N m, and 6 N m). The test setup used is shown in Fig. S4. The cell was heated to the operating temperature of 120 °C *via* heating rods inserted into the end plates. The gases used (H_2_ and air) were heated and humidified before entering the cell, and the flow rate was set to a stoichiometry of $\:\:{\uplambda}_{{\text{H}}_{2}/\text{a}\text{i}\text{r}}=\:3:2$, respectively. The polarisation measurements were conducted at a relative humidity of 20%RH and 40%RH in a temperature range between 100 and 120 °C. The current was increased stepwise by 2 mA and 10 mA, and each current was held for 2 min. To obtain a stable polarisation curve, a break-in process was also performed beforehand, in which the cell was repeatedly subjected to polarisation curve tests at 120 °C and 40%RH for a total of 50 times. The current was increased stepwise by 4 mA until a current of 20 mA was reached, followed by 10 mA steps until a voltage level of 0.1 V. Each step was held for 2 min, followed by a shutdown of the fuel cell at the end and a restart again after 10 min waiting time.

## Results and discussion

### Cell tests

**Fig. 3:**
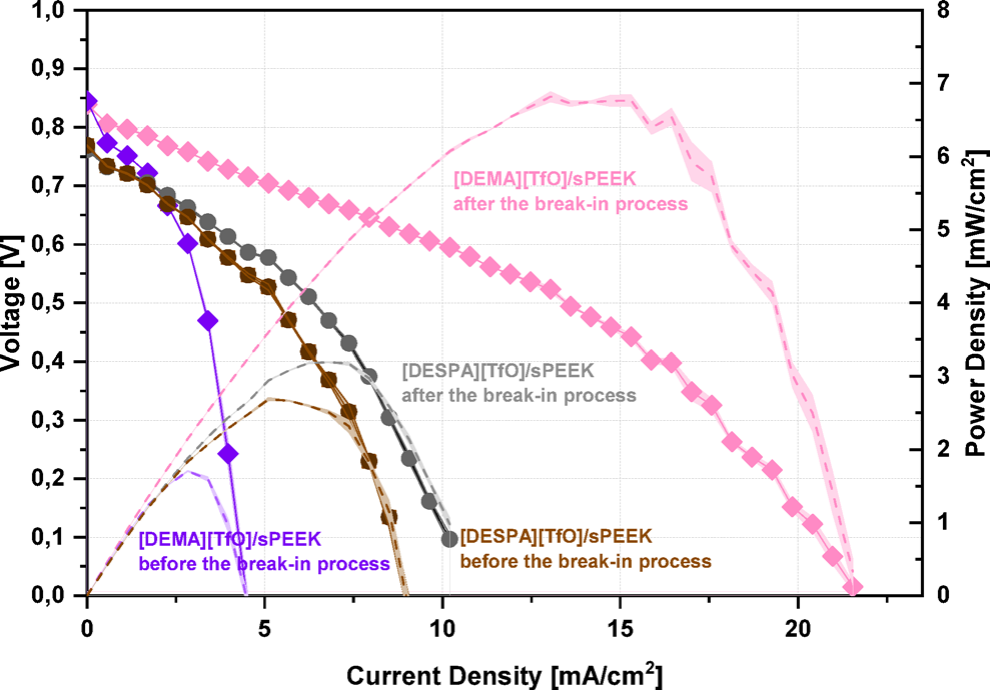
Polarisation curves of a fuel cell operating at 120 °C and 40% RH using the membrne casts [DESPA][TfO]) and [DEMA][TfO], before and after the break-in process. The symbols correspond to voltage and the dotted lines to power density. The break-in process was carried out by repeating the polarisation curves 50 times.

For the investigation of the suitability of the [DESPA][TfO]/sPEEK and [DEMA][TfO]/sPEEK composite membranes for an application as PEM for future IT-PEMFCs, test fuel cells were assembled to measure the *I vs. U* polarisation curves. The obtained polarisation curves before and after a break-in process are shown in Fig. 3. Comparing the results before and after the break-in process, the maximum output power density (depicted as dotted lines in Fig. 3) of the [DESPA][TfO]/sPEEK membranes is about 1.5-fold higher than for the [DEMA][TfO]/sPEEK membranes, 2.8 and 1.8 mW.cm^−2^, respectively.

Regarding the influence of the break-in process, overall, an improvement of the maximum power density is obtained up to 3.8-fold. The break-in process involves repeating the polarisation curve fifty times, thereby enhancing the hydration state of the membrane as sufficient time is available for impregnating the membrane with the generated water. Comparing the maximum power densities of the PIL/sPEEK membranes, the influence of the break-in process becomes especially evident. In the case of the [DEMA][TfO]/sPEEK membranes, an increase of the maximum output power density from 1.8 up to 7 mW.cm^−2^ is achieved, which is about 14-fold higher than in the case of [DESPA][TfO]/sPEEK membranes, from 2.8 to 3.2 mW.cm^−2^. The difference in the impact of the break-in process is quite interesting as in the case of [DESPA][TfO], the higher water content, as it should have after the break-in process, should further increase the conductivity compared to the neat PIL, due to more mobile protonic charge carriers (H_3_O^+^, water) available. In addition to vehicular proton transport, a cooperative mechanism is possible^32,38^. However, the conductivity measurements in the next section of this publication will give further information about the proton conduction mechanism of the PIL/sPEEK membranes. Compared to literature, however, the obtained max. power densities and current densities are magnitudes lower than for the commercially available phosphoric acid-doped PBI^39,40^.

The disparate extent of the break-in processes for the PIL/sPEEK membranes might be attributed to the loss of the conductive electrolyte, the PIL. The loss of the PIL might occur due to two mechanisms. On the one hand, the generated water during the fuel cell test could leach the PIL out of the membrane and result in a loss of the conductive electrolyte. Thereby, the amount of the PIL decreases with the duration of the break-in process as the amount of water produced increases and can drag more PIL. This would be accompanied by a decreased overall conductivity of the membrane, counteracting the improvements of the break-in process and, thus, a reduced influence of the break-in process. In the literature, PIL loss during operation can be observed as a decrease in conductivity due to high PIL loadings^41^. A further aspect leading to the loss of PIL could be a shift of the protolysis reaction, at the operating temperature, to the free acid HA and base B, leading to more volatile, respectively, thermally less stable components within the system^38^. Hence, in the case of the [DESPA][TfO]/sPEEK membrane, it could lead to more evaporation of TfOH than in the case of the [DEMA][TfO]/sPEEK membrane.

### (Total) ionic conductivity

**Fig. 4:**
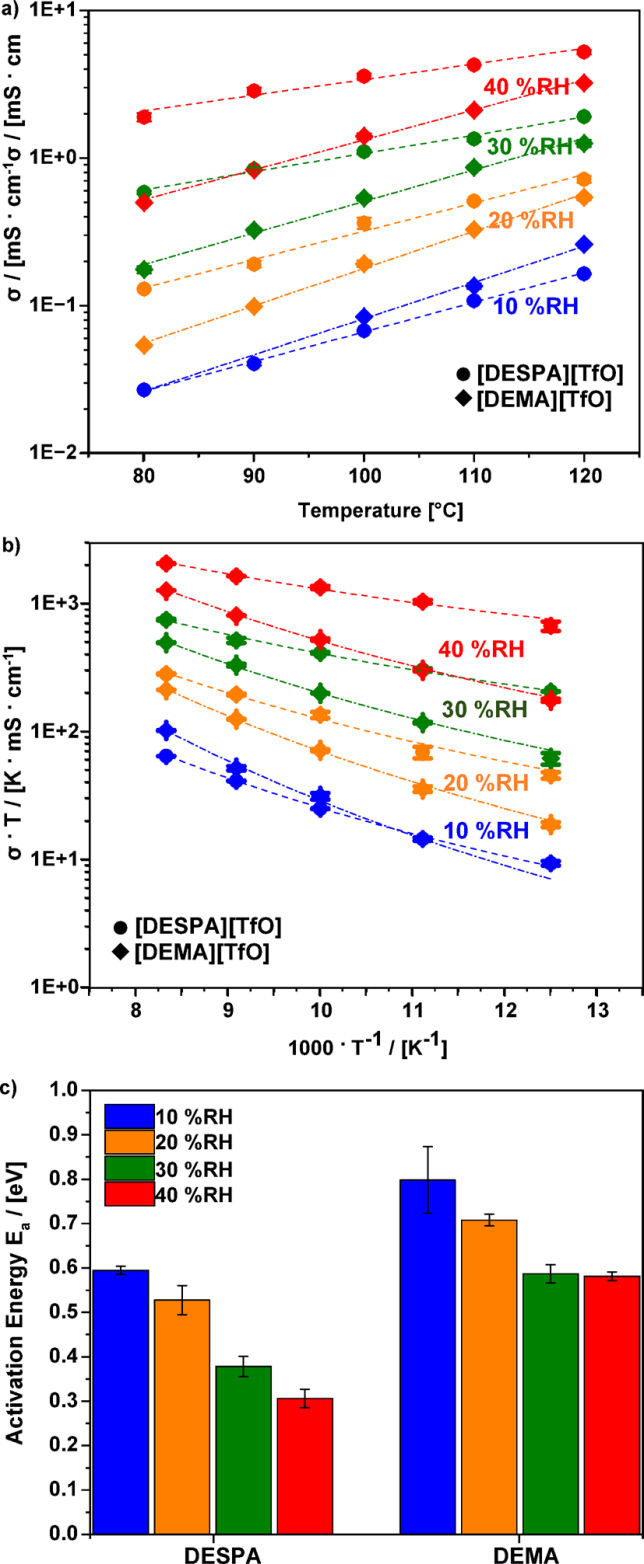
Investigation of ionic conductivities of PIL membrane casts doped with [DEMA][TfO] and [DESPA][TfO], a) Ionic conductivities of the PEMs at different relative humidity and temperatures; (b) temperature-dependence of the ionic conductivities of the PEM employing an Arrhenius plot, the dashed lines correspond to the Arrhenius fitting; (c) the activation energies of PEMs at different relative humidity, calculated from the slopes of the fitted lines.

An important property of the PIL/sPEEK membranes represents the ionic conductivity assessed at different conditions, *e.g.* variation of the water content and temperature (Fig. 4). Additionally, the influence of varying weight contents of the ionic liquids on the conductivity was also investigated, see Table S1. Thereby, three membranes with a mixture of the two ionic liquids (PEM 1 – PEM 3) with increasing ionic liquid content (49.3 wt% IL, 59.3 wt% IL and 66.0 wt% IL) within the PEM were tested in addition to the two membranes doped with the pure ionic liquids (PEM 4 and PEM 5). It can be seen that with an increasing amount of ionic liquid, the conductivity of the composite membranes increases; thus, the highest possible doping level of 66 wt% was used for the PEMs with only one ionic liquid. Figure 4a depicts the total conductivity of the PIL/sPEEK membranes measured by EIS in the temperature range of 80–120 °C and relative humidity (%RH) of 10–40%RH. The total data of the conductivity measurements is shown additionally in Fig. S6. Two features can be observed in Fig. 4a; with increasing temperature and increasing %RH, an enhancement of the conductivity is observed. This becomes especially evident by taking a closer look at the behaviour of [DESPA][TfO]/sPEEK membranes at 30%RH with increasing temperature. A more than 3-fold increase in conductivity from 0.59 mS.cm^−1^ up to 1.92 mS.cm^−1^ is obtained with increasing temperature. In the case of the [DEMA][TfO]/sPEEK membranes, the conductivity increase in the temperature dependence is even higher at 30%RH. An about 7-fold increase is obtained, from 0.18 mS.cm^−1^ up to 1.27 mS.cm^−1^. The influence of the temperature on the conductivity reflects the thermal activation of ionic conductivity. Moreover, the influence of the relative humidity on the conductivity of the two composite membranes [DESPA][TfO]/sPEEK and [DEMA][TfO]/sPEEK appear to be even more decisive than the influence of the temperature, as can be seen additionally in Fig. S5. Thereby, an up to 52-fold increase of conductivity is observed, *e.g.* at 100 °C for a change from 10 to 40%RH, [DESPA][TfO]/sPEEK and [DEMA][TfO]/sPEEK show an increase of conductivity from 0.07 mS.cm^−1^ up to 3.62 mS.cm^−1^ and 0.08 mS.cm^−1^ up to 1.41 mS.cm^−1^, respectively. Comparing the obtained results here in Fig. 4 to the obtained results of the *I vs. U* curve in Fig. 3 for the [DEMA][TfO]/sPEEK after the break-in process, an area-specific resistance of 20 Ω.cm^−2^ of the membrane with the corresponding conductivity of 1 mS.cm^−1^ can be approximated. This means that the results of the conductivity measurements in Fig. 4 correspond to the results obtained during the fuel cell tests. However, compared to phosphoric acid-doped PBI, the composite membranes have a magnitude lower conductivity^42^. While the PA/PBI membrane obtains conductivities around 13 and 17 mS.cm^−1^ at 80 °C for 20%RH and 40%RH, the composite membranes are in the range of 0.05 to 1.91 mS.cm^−1^ at the same conditions. Several mechanisms may be present to explain the observed effects. On the one hand, with increasing %RH also, the water content within the membrane is expected to be higher, which might increase the necessary viscous flow accompanied by local ion migration. Furthermore, with increasing water content, the sPEEK material supports conduction, as the sulfonic acid functional groups form hydrophilic domains in contact with water, resulting in proton conductivity similar to Nafion^®^ *via* the vehicular mechanism. Moreover, besides the cations [DEMA]^+^ and [DESPA]^+^, H_3_O^+^ might contribute to vehicular proton transportation. The higher acidity of [DESPA][TfO] enables the easier release of protons to the water molecule, resulting in more protons available for proton conduction *via* vehicle transport with H_3_O^+^. Additionally, H_3_O^+^ is smaller than the cations of the PILs, resulting in a faster diffusion velocity^38,43,44^. Furthermore, due to the higher acidity of [DESPA][TfO] and the more pronounced protolysis, an additional proton-conducting mechanism *via* the cooperative transport is present^38,45^. Thus, the interaction of the mechanisms explained leads to the observed increased conductivity as a function of %RH. Comparing the obtained conductivities of [DESPA][TfO]/sPEEK membrane to the sPEEK membrane alone, twice as high conductivity is obtained, 0.9 mS.cm^−1^and 1.91 mS.cm^−1^at 80 °C and 40%RH (DS = 50%)^46^. Additionally, comparing the conductivity of the composite membrane to the neat PIL, an up to 30-fold difference in conductivity is seen for [DESPA][TfO. The neat PIL shows conductivities in the range of 0.7 up to 0.9 mS.cm^−1^^47^, while for the [DESPA][TfO]/sPEEK membrane, the conductivities are in the range of 0.03 up to 0.2 mS.cm^−1^ (T = 80 to 120 °C for both). Thus, the neat PIL shows a much higher conductivity than immobilised within the composite membrane, further underlining the incompatibility between sPEEK and the PIL.

Comparing the conductivities of the two composite membranes [DESPA][TfO]/sPEEK and [DEMA][TfO]/sPEEK in dependence of temperature and %RH, an inverse effect becomes apparent. The conductivity of [DEMA][TfO]/sPEEK exhibits a higher dependence on the temperature, while the conductivity of [DESPA][TfO]/sPEEK is dependent on the relative humidity. To explain this inversion, the activation energies for ionic conduction of the two PEM casts $\:{E}_{a}$ are investigated, which indicate the energy required for the rate-limiting step of the conduction process^48^. Fig. 4b and c show the Arrhenius plot of the composite membranes [DESPA][TfO]/sPEEK and [DEMA][TfO]/sPEEK and the associated $\:{E}_{a}$ with the standard errors. When considering Fig. 4b and c, two notable features are present. On the one side, the activation energies of the membranes [DESPA][TfO]/sPEEK and [DEMA][TfO]/sPEEK exhibit a decreasing trend with increasing %RH. Thereby, a decrease of up to 50% is obtained, *e.g.* [DEMA][TfO]/sPEEK shows a decrease from 0.8 eV at 10%RH to 0.6 eV at 40%RH, while [DESPA][TfO]/sPEEK shows a decrease from 0.6 eV at 10%RH to 0.3 eV at 40%RH. Comparing the absolute activation energies of the membrane casts in Fig. 4(c), it becomes evident that [DESPA][TfO]/sPEEK shows a smaller activation energy than [DEMA][TfO]/sPEEK at the same relative humidity. For example, at 30%RH [DESPA][TfO]/sPEEK shows a 1.5 times lower activation energy than [DEMA][TfO]/sPEEK, 0.4 eV and 0.6 eV, respectively. Therefore, the previously discussed hypothesis regarding the decline in activation energy with increasing %RH enabling a higher ionic conductivity in dependence of the %RH is verified. In detail, several mechanisms are present; the increase of the %RH, on the one hand, might increase the necessary local viscous flow, which, in combination with an increased proportion of proton transport *via* H_3_O^+^, leads to a reduction of the overall energy barrier for vehicular proton transport. Moreover, the higher proportion of proton transport *via* H_3_O^+^ instead of the individual cation is a result of the higher acidity of [DESPA][TfO], leading to more protons available for the proton conduction *via* vehicle transport with H_3_O^+^ within the membrane cast containing [DESPA][TfO], leading to a lower activation energy. Additionally, with the increasing %RH also, a cooperative mechanism might contribute to the proton conductivity as well^38^.

Different magnitudes are present, comparing the activation energies for the respective PIL/sPEEK membranes at the same %RH. For example, at 30%RH, [DESPA][TfO]/sPEEK exhibits a 1.6-fold lower activation energy than [DEMA][TfO]/sPEEK, 0.38 eV and 0.59 eV, respectively. Different molar fractions of the respective PILs might explain this effect. Even though the mass fractions within the membrane casts are the same for both [DEMA][TfO] and [DESPA][TfO], 66.0 wt%. However, [DEMA][TfO] has a lower molecular weight than [DESPA][TfO], resulting in a higher molar fraction at a low %RH of 10%. Furthermore, a higher absolute conductivity of the membrane cast with [DEMA][TfO] is obtained, as a more significant molar fraction is available for ionic conduction. The bulk conductivity of the respective PILs is, in the case of [DEMA][TfO], up to 2.5-fold higher than for [DESPA][TfO], at low %RH 0.02 S.cm^−1^and 0.008 S.cm^−1^, respectively^32,47^. At higher %RH, the importance of the protolysis from the sulfonic acid groups of [DESPA]^+^ cations to the water molecules is increased, leading to a significant enhancement of the conductivity in the case of the PIL membrane cast with [DESPA][TfO] with increasing %RH. Thus, increasing temperatures and increasing %RH lead to an enhancement of the conductivity of the investigated composite membranes.

### Thermogravimetric characterisation

**Fig. 5:**
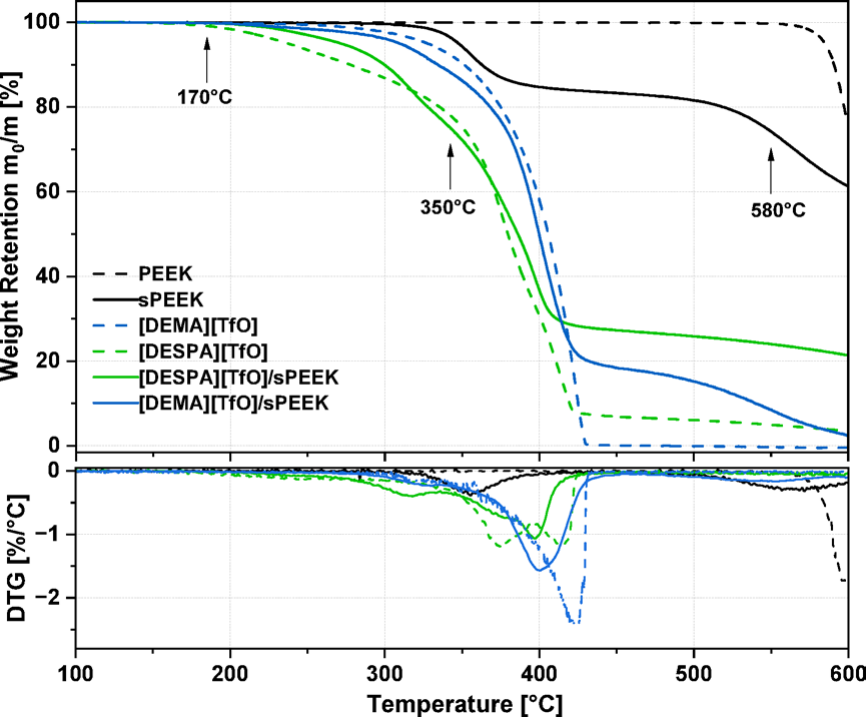
Thermal Analysis; (a) TGA and (b) DTG curves of PEEK, sPEEK, [DEMA][TfO], [DESPA][TfO], and PIL/membrane casts.

For the elucidation of the observed effects during the cell tests and to verify the suitability of PILs as conducting electrolytes within a sPEEK host polymer, [DEMA][TfO] and [DESPA][TfO] PILs and derived membranes were investigated *via* TGA, EIS and SEM (see below). The thermal gravimetric analysis (TGA) of the host polymer before (PEEK), after the sulfonation process (sPEEK), of the two pristine PILs [DEMA][TfO] and [DESPA][TfO], as well as of the PIL/sPEEK composite membranes, are shown in Fig. 5(a). The derivative thermogravimetric analysis (DTG) curves are depicted in Fig. 5(b). The DTG curves represent the first derivative of the weight retention curves shown in Fig. 5(a), *i.e.* the inflection point in the TGA curve represents the peaks of the DTG curve, indicating the rate of weight change. The thermic analysis depicted in Fig. 5(a) reveals a single weight loss step at 580 °C for the PEEK polymer, which describes the starting point of the polymer backbone decomposition^49^. Comparing the non-sulfonated (PEEK) and sulfonated host polymer (sPEEK), an additional weight loss step occurs at 350 °C for sPEEK. This additional weight-loss step is due to the loss of the sulfonic acid groups (-SO_3_H), *i.e.* a thermal desulfonation occurs^50^. Thus, according to the observed decomposition steps > 300 °C, the host polymer (sPEEK) exhibits sufficient thermal stability for application in the IT-PEMFC.

Comparing the thermal stability of the neat PILs, [DEMA][TfO] exhibits a higher thermal stability than [DESPA][TfO]. The higher thermal stability is defined by the higher onset temperature for the weight loss. In the case of [DEMA][TfO], a value of 350°C^51^ can be measured, which is significantly higher compared to the value found for [DESPA][TfO] of 170 °C^47^. A possible explanation for the different thermal stabilities of the investigated PILs is probably the degree of the protolysis reaction between the anion precursor acid HA to the cation precursor base B, leading to thermally stable ionic liquids [HB][A]:

6 $${\text{HA }} + {\text{ B}} \rightleftarrows {\text{HB}}^{ + } + {\text{ A}}^{ - }$$

In a first approximation, neglecting the validity of the Brønsted acidity constants only for diluted aqueous systems, the equilibrium constant of the protolysis in Eq. (6) can be expressed as the ratio $\:{K}_{a,\text{H}\text{A}}/{K}_{a,{\text{H}\text{B}}^{+}}$ of the acidity constants of the conjugated acid HA of the anion and the cation HB^+^ Eq. (7):

7 $$\:\:{K}_{a,\text{H}\text{A}}\cdot\:\frac{1}{{K}_{a,{\text{H}\text{B}}^{+}}}=\frac{{a}_{{\text{A}}^{-}}\:{a}_{{\text{H}}_{3}{\text{O}}^{+}}}{{a}_{\text{H}\text{A}}\:{a}_{{\text{H}}_{2}\text{O}}}\cdot\:\frac{{a}_{{\text{H}\text{B}}^{+}}\:{a}_{{\text{H}}_{2}\text{O}}}{{a}_{\text{B}}\:{a}_{{\text{H}}_{3}{\text{O}}^{+}}}=\frac{{a}_{{\text{H}\text{B}}^{+}}\:{a}_{{\text{A}}^{-}}}{{a}_{\text{H}\text{A}}\:{a}_{\text{B}}}$$

PILs with a large ratio $\:{K}_{a,\text{H}\text{A}}/{K}_{a,{\text{H}\text{B}}^{+}}$, respectively with a large difference $\:\varDelta\:\text{p}{K}_{a}={\text{p}K}_{a,\text{H}\text{A}}-{\text{p}K}_{a,{\text{H}\text{B}}^{+}}$, suggest a complete proton transfer from HA to B. The $\:\varDelta\:\text{p}{K}_{a}$of [DEMA][TfO] has a value of 17.5^19^, indicating good thermal stability, as a $\:\varDelta\:\text{p}{K}_{a}\:$≥ 15 prevails against back protonation and is explaining its higher thermal stability^52^. Furthermore, [DESPA][TfO] shows 2 weight loss steps, a first step starting at 170 °C and a second one at 350 °C. A possible explanation of these two weight loss steps instead of just one, as observed for [DEMA][TfO], can be a re-protonation process at a considerably low temperature^51^. Therefore, with increasing temperature a shift of the protolysis equilibrium to the pristine acid HA and base B is expected, leading to the presence of more volatile, respectively thermally less stable components within the system^38^. This explains the first weight loss step, the evaporation of triflic acid TfOH with a boiling point of 162 °C^53^. Hence, this could explain the PIL loss of [DESPA][TfO] during the break-in process leading to the disparate extent of the break-in process. In the case of [DESPA][TfO], the second weight loss step, starting at 350 °C, is most probably due to the thermal decomposition of the free base diethylsulfopropylamine, starting with a desulfonation. In the solid phase it is present as a zwitterion and thus it is only less volatile^38^.

The PIL/sPEEK membranes are also investigated regarding their thermal stability. Comparing the individual PILs to the respective PIL/sPEEK membranes, it is noticeable that the thermal properties of the individual PIL are retained, respectively. The onset temperature of the weight loss remains the same, as does the extent of the thermal stability, depending on the respective pristine PIL. Thereby, the [DEMA][TfO]/sPEEK membranes again show higher thermal stability than the [DESPA][TfO]/sPEEK membranes. Overall, the thermal stability of sPEEK is decreased due to the incorporation of PILs, whereby the extent depends strongly on the incorporated PIL. Concluding, the TGA measurements verified the thermal stability of the PIL/sPEEK membranes at the intended operating temperature of an IT-PEMFC.

### Structural investigation by SEM

**Fig. 6:**
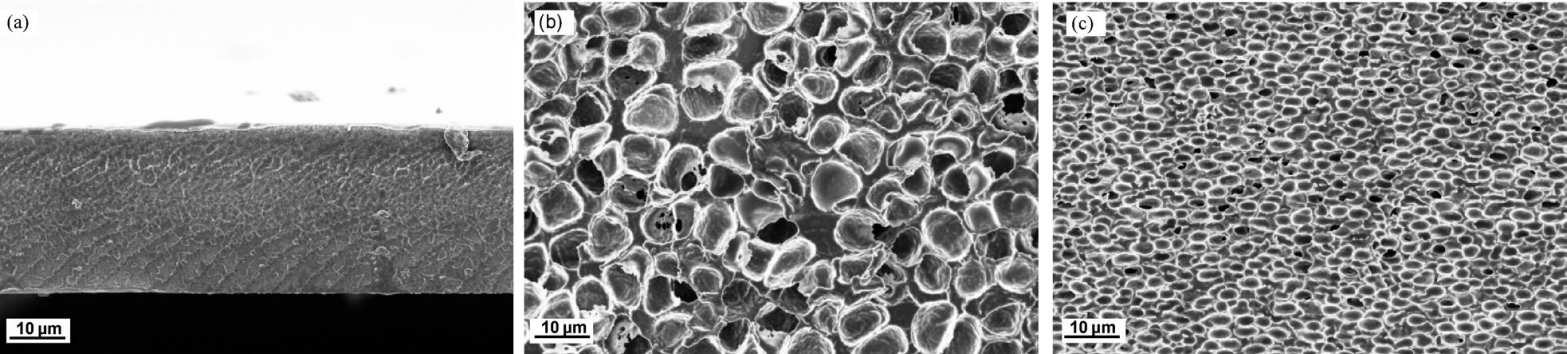
SEM images of the cross sections of (a) pristine sPEEK, (b) [DEMA][TfO]/sPEEK membrane and (c) [DESPA][TfO]/sPEEK membrane.

To elucidate the visual appearance of the membrane’s cross-sections and to determine the hypothesis of PIL loss, SEM images of the pure sPEEK membrane and the respective doped membranes with [DEMA][TfO] and [DESPA][TfO] are depicted in Fig. 6(a)-(c). The pure sPEEK membrane shows a uniform and compact microstructure; see Fig. 6(a). Meanwhile, in the case of composite membranes, Fig. 6(b)-(c), no continuous layer is present, and a phase separation occurs. Doping the sPEEK membrane with ionic liquids thereby resulted in spherical-shaped PIL droplets within the membrane, forming a sponge-like structure, which might occur due to the incompatibility of the components. The obtained spherical PIL droplets are probably the reason for the distinctive difference in the break-in process for the [DESPA][TfO]/sPEEK membrane, as they could lead to easy PIL loss with the produced water due to their non-homogeneity or due to the applied pressure during assembly. While no pressure was applied during the EIS measurements, it showed the “true conductivity” obtained with the PIL/sPEEK membranes. Furthermore, with increasing %RH, the polymeric matrix additionally became conductive. This effect is further enhanced in the [DESPA][TfO]/sPEEK membrane due to the protolysis of the PIL, possibly explaining the lesser extent of the break-in process^54^.

## Conclusion

In this study, composites of protic ionic liquids and ionogene polymers as host matrices are investigated for use in future fuel cells operating above 100 °C (IT-PEMFC). Membranes were prepared using sPEEK as the host polymer and two commercially available PILs [DESPA][TfO] and [DEMA][TfO]. During the fuel cell measurements, different impacts of a break-in process were observed on the two PIL/sPEEK composite membranes. While the [DESPA][TfO]/sPEEK membranes showed before the break-in process a 1.5-fold higher maximum power density than the [DEMA][TfO]/sPEEK membranes, after the break-in process, the result was reversed. The [DEMA][TfO]/sPEEK membranes showed thereby a 14-fold higher maximum power density than [DESPA][TfO]/sPEEK membranes. A possible reason for this change was the loss of conducting electrolyte revealed due to the non-homogeneous structure, and possible mechanical damage due to the assembly pressure. A striking information obtained is their good thermal stabilities until 150 °C, establishing sufficient stability for IT-PEMFCs with an operating temperature of 100–120 °C. In comparison with the individual constituents, the thermal stability of the membranes was highly dependent on the component with the lowest stability, the PILs, due to a possible back protonation process at high temperatures, leading to the evaporation of the most volatile precursor component, TfOH, before a complete decomposition of the polymer occurs. This feature is especially evident for PILs with low $\:\varDelta\:\text{p}{K}_{\text{a}}$, explaining the lower thermal stability of the [DESPA][TfO]/sPEEK membranes.

Elucidating the conductivity of the PIL/sPEEK composite membranes, EIS measurements revealed a dependence of the temperature and %RH on the conductivity. Elevated temperatures decrease the required activation energy for the ionic transport, and an increased relative humidity leads to an increased hydration state of the membrane and additional ionic conduction through a cooperative mechanism. Furthermore, [DESPA][TfO]/sPEEK membranes exhibit good conductivity up to 5.28 mS.cm^−1^ at 120 °C and 40%RH. The activation energy analysis indicates that [DESPA][TfO]/sPEEK PEMs possess a low activation energy, especially at a higher relative humidity. This might be related to the fact that cation [DESPA]^+^ can easily release a proton to facilitate the additional cooperative proton transport *via* H_3_O^+^. In conclusion, the [DESPA][TfO]/sPEEK membrane is, in theory, a promising candidate for a future IT-PEMFC due to its high conductivity. However, for use in fuel cells, the loss of the conducting electrolyte [DESPA][TfO] due to the non-homogenous membrane structure needs to be resolved in advance.

## Supplementary Information

Below is the link to the electronic supplementary material.

Supplementary Material 1

## Data Availability

The authors declare that the data supporting the findings of this study are available within the paper and its Supplementary Information files. Should any raw data files be needed in another format they are available from the corresponding author upon reasonable request.
