# Supplementary material for: Proton-conducting polymer electrolyte membranes based on sulfonated PEEK and blends of protic ionic liquids with different acidities
Source: Sci Rep. 2025 Oct 9;15:35234. doi: 10.1038/s41598-025-22147-3 (PMC12511278; doi:10.1038/s41598-025-22147-3)
Supplement: Supplementary file 1 — Supplementary Material 1 [file 41598_2025_22147_MOESM1_ESM.docx]

**Supplementary Information**

**Proton-conducting polymer electrolyte membranes based on sulfonated PEEK and blends of protic ionic liquids with different acidities.**

V. Theußl,^a^ X. Cui,^a^ H. Hou,^a^ A. Gueguen,^c^ C. Rodenbücher,^a^ C. Korte,*^a,b^

*^a^Institute of Energy Technologies – Electrochemical Process Engineering (IET-4), Forschungszentrum Jülich GmbH, 52425 Jülich, Germany*

*^b^RWTH Aachen University, Institute of Physical Chemistry, Landoltweg 2, 52074 Aachen, Germany*

*^c^ Toyota Motor Europe BV Technical Centre, Hoge Wei 33, 1930 Zaventem, Belgium*

*Corresponding author’s email address: c.korte@fz-juelich.de


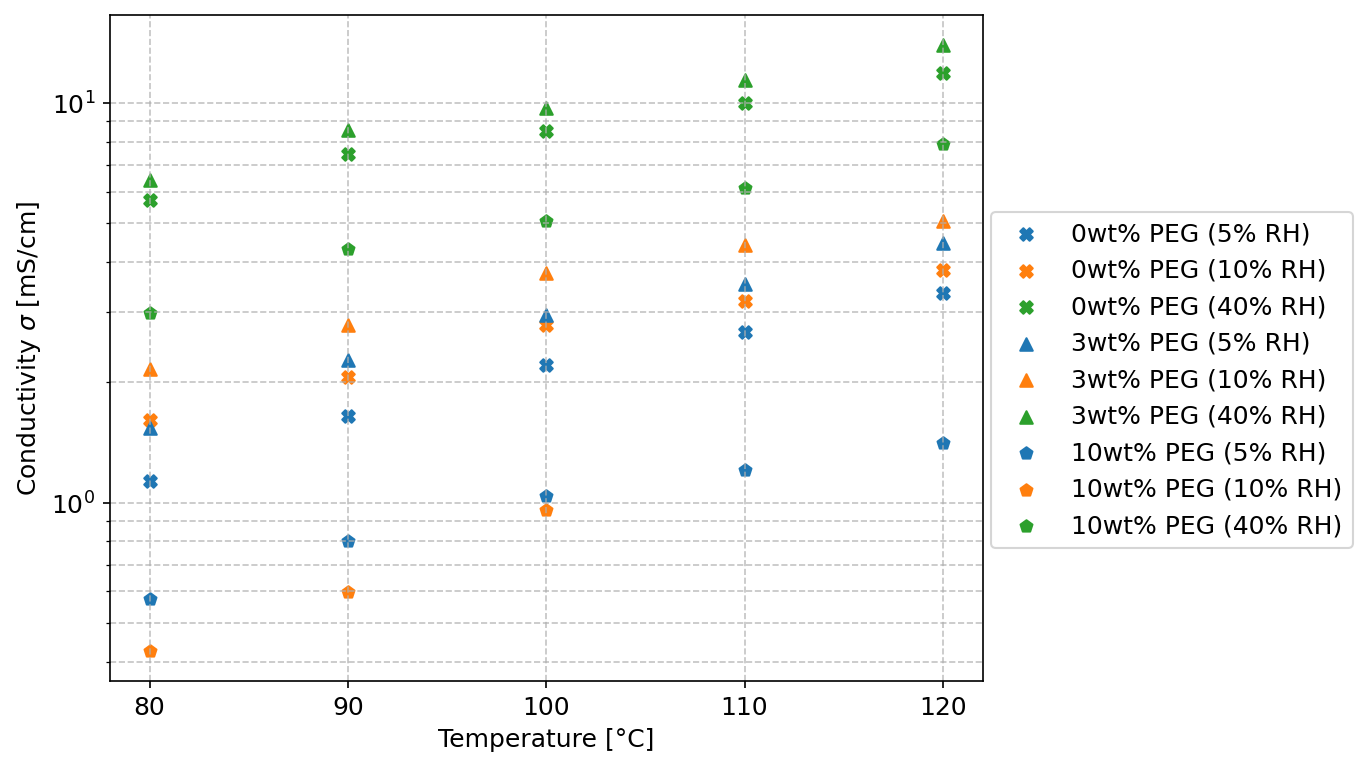


**Figure S 1:** Influence of PEG content (0 – 10 wt%) on the ionic conductivity at different humidities (5 – 40%RH). For testing, a mixed PIL (1:1 m([DESPA][TfO]):m([DEMA][TfO] was employed with a sPEEK amount of 2:1. The mass percentage of PEG in the legend is relative to the mass of sPEEK polymer.


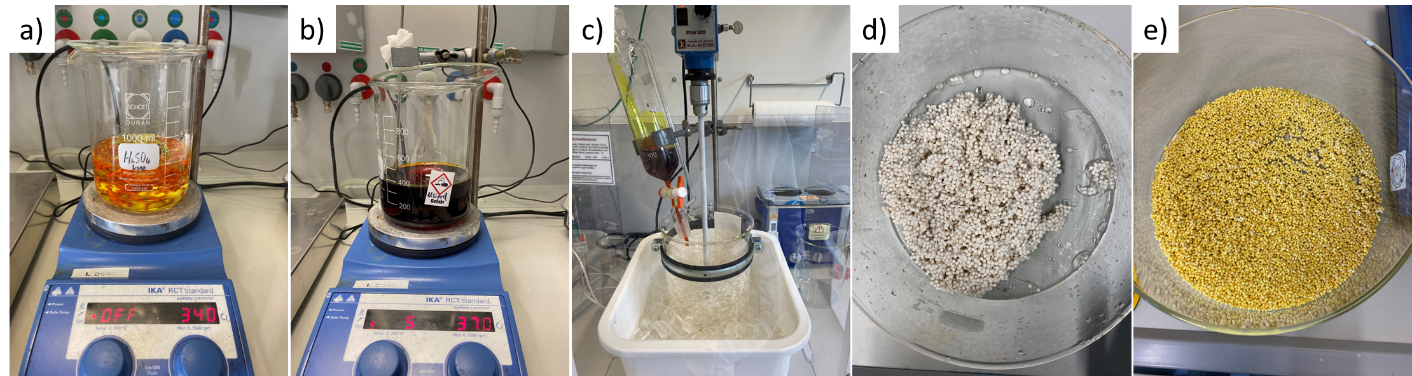


**Figure S 2:** Process of PEEK sulfonation, a) PEEK pellets in H_2_SO_4_ at the beginning of dissolving; b) dissolved PEEK in H_2_SO_4_ during sulfonation; c) SPEEK precipitation process; d) SPEEK precipitates before drying; e) SPEEK particles after drying.

**Figure S 3**: Temperature program TGA for the determination of the thermal stability of the investigated membranes.


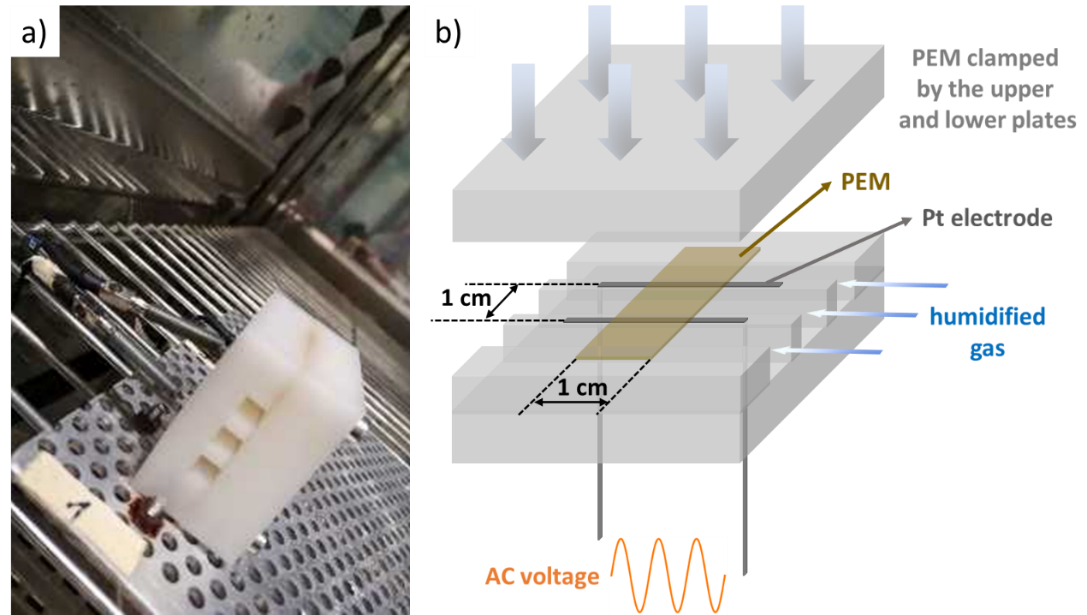


**Figure S 4:** Conductivity measurements, (a) picture of test cell in the climate chamber, (b) schematic depiction of the test cell.


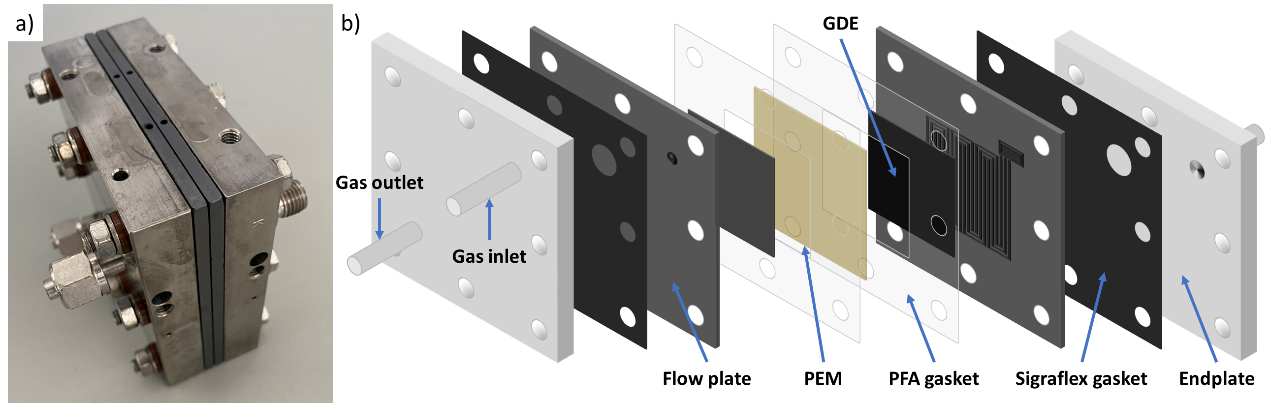


**Figure S 5:** Electrochemical investigation of the membranes, (a) Picture of the test cell rig and (b) schematic depiction of the single test cell for IT-PEMFC.


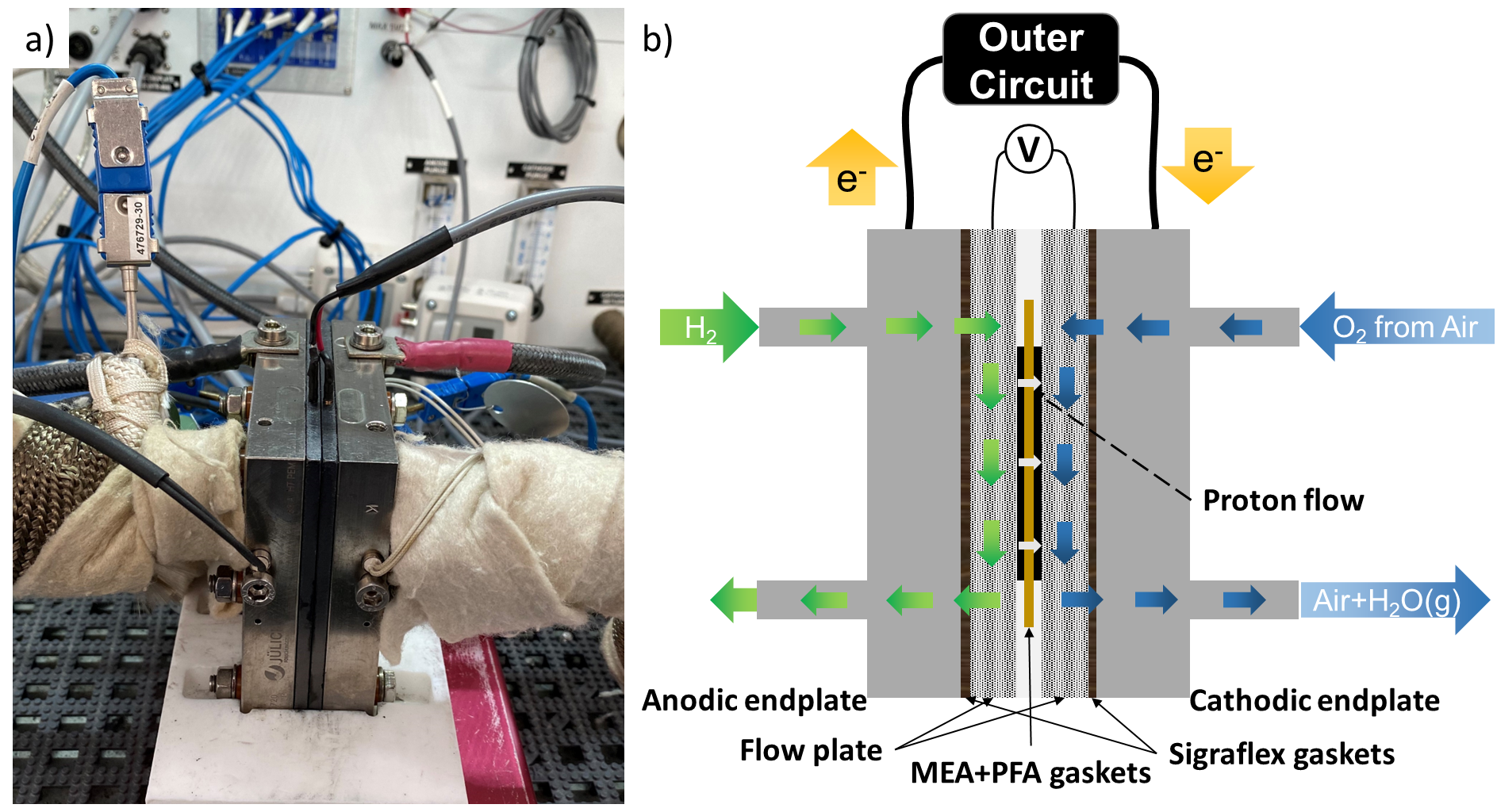


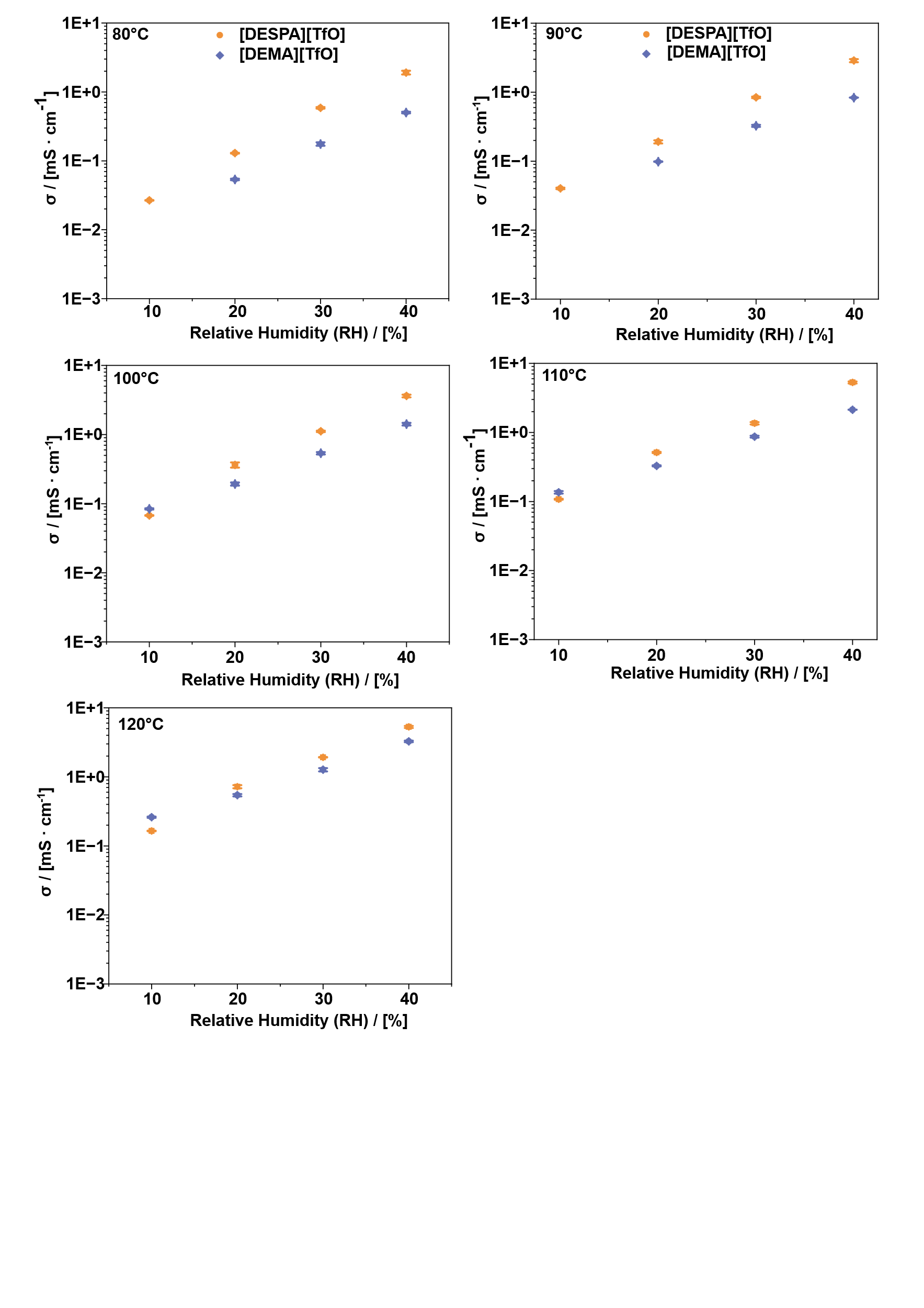


**Figure S 6:** Influence of humidification on the conductivity of the composite membranes sPEEK/[DEMA][TfO] and sPEEK/[DESPA][TfO].

**Table S 1**:Influence of the type and content of ionic liquid on the conductivity. PEM 1 - 3 are mixtures of [DESPA][TfO] and [DEMA][TfO] in different concentrations (49.3 wt% IL, 59.3 wt% IL and 66.0 wt% IL), where the amount of [DESPA][TfO] and [DEMA][TfO] were the same. PEM 4 (66.0 wt% [DESPA][TfO]) and PEM 5 (66.0 wt% [DEMA][TfO]) are the respective pure ionic liquids incorporated into the sPEEK polymer matrix.

| **Condition** |  | **Conductivity σ [mS/cm]** |  |  |  |  |
| --- | --- | --- | --- | --- | --- | --- |
| **R.H.** | **Temp.** | **PEM1** | **PEM2** | **PEM3** | **PEM4** | **PEM5** |
| **10%** | **80 °C** | 0.08 | 0.12 | 0.15 | 0.03 | 0.15 |
|  | **90 °C** | 0.15 | 0.17 | 0.21 | 0.04 | 0.17 |
|  | **100 °C** | 0.2 | 0.22 | 0.26 | 0.07 | 0.08 |
|  | **110 °C** | 0.24 | 0.25 | 0.34 | 0.11 | 0.14 |
|  | **120 °C** | 0.3 | 0.28 | 0.45 | 0.16 | 0.26 |
| **20%** | **80 °C** | 0.18 | 0.19 | 0.27 | 0.13 | 0.05 |
|  | **90 °C** | 0.25 | 0.26 | 0.35 | 0.19 | 0.1 |
|  | **100 °C** | 0.32 | 0.35 | 0.49 | 0.36 | 0.19 |
|  | **110 °C** | 0.39 | 0.44 | 0.6 | 0.51 | 0.33 |
|  | **120 °C** | 0.49 | 0.55 | 0.77 | 0.72 | 0.54 |
| **30%** | **80°C** | 0.36 | 0.46 | 0.57 | 0.59 | 0.18 |
|  | **90°C** | 0.46 | 0.63 | 0.78 | 0.84 | 0.33 |
|  | **100°C** | 0.6 | 0.91 | 1.03 | 1.11 | 0.54 |
|  | **110°C** | 0.74 | 1.09 | 1.34 | 1.36 | 0.87 |
|  | **120 °C** | 0.99 | 1.58 | 1.56 | 1.92 | 1.27 |
| **40%** | **80°C** | 0.76 | 1.21 | 1.24 | 1.91 | 0.5 |
|  | **90°C** | 0.98 | 1.65 | 1.66 | 2.87 | 0.83 |
|  | **100°C** | 1.28 | 2.37 | 2.4 | 3.62 | 1.41 |
|  | **110°C** | 1.6 | 3.1 | 3.09 | 4.31 | 2.13 |
|  | **120 °C** | 1.93 | 3.66 | 4.12 | 5.28 | 3.26 |
